# Genome-Wide Identification and Expression Analysis of the Melon B-BOX (BBX) Gene Family in Response to Abiotic and Biotic Stresses

**DOI:** 10.3390/plants14172715

**Published:** 2025-09-01

**Authors:** Yu Zhang, Yin Li, Yan Wang, Congsheng Yan, Dekun Yang, Yujie Xing, Xiaomin Lu

**Affiliations:** 1 College of Agriculture, Anhui Science and Technology University, Fengyang 233100, China; 2 Institute of Vegetables, Anhui Academy of Agricultural Sciences, Hefei 230001, China; 3 Key Laboratory of Horticultural Crop Germplasm Innovation and Utilization (Co-Construction by Ministry and Province), Hefei 230001, China; 4 Anhui Provincial Key Laboratory for Germplasm Resources Creation and High-Efficiency Cultivation of Horticultural Crops, Hefei 230001, China

**Keywords:** BBX gene family, melon, biotic and abiotic stresses, RNA-seq

## Abstract

The BBX gene family functions as a key transcription factor implicated in plant growth, development, and stress responses. However, research on this gene family in melon remains absent. In the present study, we identified 19 BBX family genes within the melon genome, distributed across chromosomes 1, 2, 3, 4, 5, 7, 8, 10, 11, and 12. Phylogenetic analysis categorized these genes into five distinct subfamilies, with notable similarities observed in gene structure and conserved motifs among members of the same subfamily. Synteny analysis revealed seven syntenic relationships among melon BBX genes, 17 between melon and *Arabidopsis*, and one between melon and rice. Reanalysis of transcriptome data indicated that certain BBX genes exhibit high expression levels across various tissues and developmental stages of fruits, while others display tissue specificity. Under both abiotic and biotic stress conditions, genes such as *CmBBX3*, *CmBBX5*, *CmBBX2*, *CmBBX18*, *CmBBX15*, and *CmBBX11* demonstrated significant differential expression, highlighting their critical roles in melon growth and development. Additionally, RT-qPCR analysis was conducted to examine the expression levels of melon BBX genes at different time points under salt stress, further validating the transcriptome data. This study provides a theoretical foundation for future molecular breeding efforts in melon.

## 1. Introduction

Transcription factors (TFs) have emerged as a focal point in the realm of plant molecular biology [1]. Numerous transcription factors have been identified in higher plants, with the Plant Transcription Factor Database (Plant TFDB v5.0) cataloging a total of 320,380 TFs across 165 plant species [2]. Zinc finger protein transcription factors (ZFPs), characterized by their zinc finger domains that incorporate zinc ions, represent a substantial subgroup of transcription factors. They primarily regulate transcription, RNA packaging, and various processes through their interactions with DNA, RNA, and proteins. ZFPs play significant roles in diverse plant functions, including morphogenesis, growth and development, photosynthesis, and responses to environmental stimuli [3]. Based on structural differences, zinc finger protein transcription factors are classified into several subfamilies, including BBX, C2H2, GATA, YABBA, and WRKY gene families [4].

The B-Box (BBX) zinc finger protein constitutes a subfamily within the zinc finger protein family and has garnered significant attention due to its multifaceted functions [5]. This protein type features a BBX domain at the N-terminus, characterized by the sequence C-X2-C-X8-C-X7-C-X2-C-X4-H-X8-H; some BBX family members additionally possess a CCT domain at the C-terminus [6]. Since its initial identification in *Arabidopsis* in 1995, the biological roles of BBX genes have been extensively investigated [7]. Numerous studies have reported the presence of BBX family genes across various plant species, highlighting their positive contributions to growth, development, and stress responses. For instance, research on BBX gene families has been documented in *Arabidopsis* [8], rice [9], cucumber [10], soybean [11], watermelon [12], tomato [13], moso bamboo [14], and tartary buckwheat [15], among others. The number of BBX family genes varies across species, with each gene fulfilling specific biological functions. In *Arabidopsis*, *BBX24*/*STO* functions as a pivotal negative regulator within the UV-B signaling pathway. Quantitative reverse transcription PCR (qRT-PCR) analyses indicate that UV-B exposure significantly upregulates *BBX1*, *BBX7*, *BBX20*, *BBX25*, and *BBX32*, suggesting that these genes may represent novel components of the UV-B signaling pathway [8]. In rice, the overexpression of *OsBBX14* markedly increases the plant’s sensitivity to light, resulting in dwarfism; conversely, the knockout of *OsBBX14* leads to significantly taller plants compared to the wild type. Chromatin immunoprecipitation sequencing (ChIP-seq) and luciferase complementation imaging (LCI) assays demonstrated that this gene binds to the T/G-box of the HY5L1 promoter and interacts with *OsCRY2* (Cryptochromes), indicating that *OsBBX14* may either directly or indirectly regulate the expression of cell wall-related genes [16]. Furthermore, overexpression of *CmBBX22* in chrysanthemum substantially diminishes the plant’s drought tolerance, with transcriptomic analyses revealing that *CmBBX22* negatively regulates stomatal conductance and antioxidant responses in chrysanthemum [17]. In rapeseed, the homologous *BBX24* genes, *Bn STO*-1 and *Bn STO-2*, exhibit significant expression variations under high temperature and drought conditions [18].

Melon (*Cucumis melo* L.), a member of the Cucurbitaceae family and the Cucumis genus, is one of the most widely cultivated horticultural crops globally and serves as a model organism for investigating fruit ripening in gourd crops [19]. Advances in technology have led to continuous updates in the genome sequencing of melon. Notably, Castanera et al. [20] released the DHL92 V4 version of the melon genome in 2019, which not only addressed previous limitations but also facilitated research efforts. Consequently, an increasing number of transcriptome sequencing studies based on melon genomic data have been published, significantly advancing basic research on melons [21]. Transcriptome sequencing, also referred to as RNA-seq, is a versatile technique that enables direct analysis of transcriptomes across a wide range of organisms [22]. This method has facilitated the identification of numerous genes responsive to stress conditions, allowing for the exploration of their molecular mechanisms, thus becoming a commonly employed approach among researchers [23]. For instance, Ling et al. [24] utilized transcriptome sequencing to elucidate the resistance mechanisms of melons to downy mildew, identifying differentially expressed genes such as *FMO*, *FER*, *HD-ZIP*, and *AtHB7* under downy mildew stress. Therefore, further exploration based on the currently available melon transcriptome data can lead to the discovery of additional beneficial genes, providing valuable insights for plant molecular breeding. Recently, an increasing number of researchers have focused on plant gene families, with identified families in melons including ALDH [25], ARF [26], ACS [27], and TGA [28]. However, there have been no relevant reports regarding the BBX gene family in melons. In this study, we identified 19 BBX genes within the melon genome. Comprehensive analyses of their physicochemical properties, chromosome localization, phylogenetic relationships, and collinearity were performed. Additionally, in conjunction with published transcriptome data, the expression patterns of these 19 BBX genes across different tissues and under various stress conditions in melons were examined. The results of this study address the gap in the identification of the BBX gene family in melons and provide promising candidate genes for the molecular genetic breeding of melons to enhance resistance to multiple stresses.

## 2. Results

### 2.1. Identification of BBX Genes in Melon

In this study, the HMM file PF00643 was integrated with the published melon genome data (DHL92 V4), employing bioinformatics methods to identify 19 BBX family genes within the melon genome. The coding sequence (CDS) lengths of these genes varied from 393 bp for *CmBBX2* to 1476 bp for *CmBBX14*. Among these, *CmBBX14* encoded the largest number of amino acids (491 aa), while *CmBBX2* encoded the smallest (130 aa). The molecular weights ranged from 14.57 kDa for *CmBBX2* to 54.32 kDa for *CmBBX14*. The theoretical isoelectric points of the 19 BBX genes spanned from 4.35 (*CmBBX10*) to 8.35 (*CmBBX7*). Stability analysis indicated that, except for *CmBBX2*, which was classified as a stable protein (instability coefficient < 40), the remaining BBX proteins were considered unstable. The lipid coefficient varied between 52.66 (*CmBBX10*) and 85.85 (*CmBBX9*). Hydrophobicity analysis revealed that all 19 melon BBX genes exhibited an average hydrophobicity of less than 0, indicating that they are hydrophilic proteins. Subcellular localization predictions suggested that 15 BBX genes were localized in the nucleus, while 4 genes were predicted to be located outside the cell (Table 1).

### 2.2. Chromosomal Location of CmBBXs

**Figure 1:**
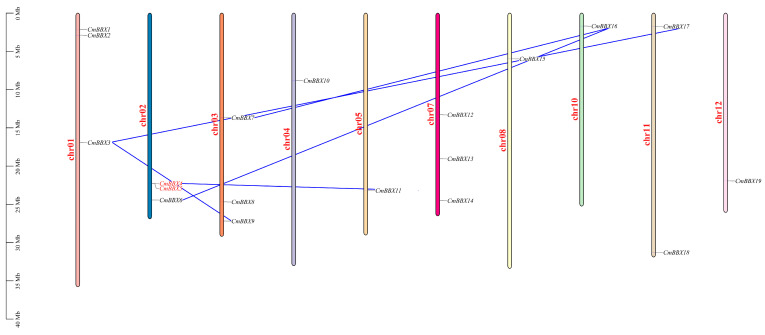
The chromosomal locations of *CmBBXs*.

Using the chromosomal localization data of the *CmBBXs*, a distribution map was generated (Figure 1). The analysis indicates that, with the exception of chromosomes 6 and 9, all other chromosomes contain BBX genes. Specifically, chromosomes 1, 2, 3, and 7 exhibit the highest gene density, each harboring three genes. Chromosome 11 contains two genes, while chromosomes 4, 5, 8, 10, and 12 have the lowest distribution, with only one gene present on each. Most genes were concentrated at the chromosomal ends, although some, including *CmBBX3*, *CmBBX7*, and *CmBBX12*, were positioned centrally. Among the 19 BBX family genes, there was one pair of tandemly repeated genes (*CmBBX4*/*CmBBX5*) and five pairs of fragmentally repeated genes (*CmBBX3*/*CmBBX9*, *CmBBX3*/*CmBBX17*, *CmBBX4*/*CmBBX11*, *CmBBX6*/*CmBBX16*, *CmBBX7*/*CmBBX16*).

### 2.3. Phylogenetic Tree Analysis of BBX Family Genes

**Figure 2:**
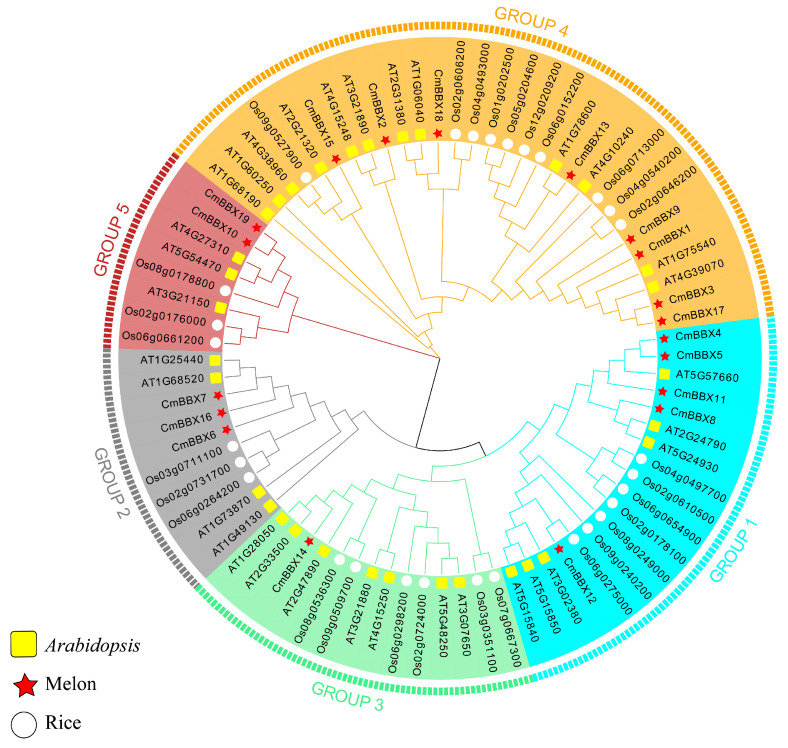
Phylogenetic relationships of melon, rice, and *Arabidopsis* BBX proteins.

Cluster analysis facilitates a comprehensive understanding of genetic relationships and biological functions among genes. This section aimed to elucidate the genetic relationships and associated biological functions of the BBX family genes in melon by comparing them with the BBX genes from the model organisms *Arabidopsis* and rice. This was achieved through multiple sequence alignment and subsequent phylogenetic tree construction and enhancement (Figure 2). Based on the classification of the BBX gene family in *Arabidopsis* and rice, the phylogenetic tree was categorized into five subgroups: GROUP1, GROUP2, GROUP3, GROUP4, and GROUP5. Notably, GROUP4 contained the highest number of melon BBX genes, totaling eight, while GROUP1 had five, and GROUP3 included the fewest, with only one gene. The analysis identified a pair of orthologous genes (*CmBBX13*/*AT1G78600*) between melon and *Arabidopsis* BBX family genes, as well as another pair (*CmBBX12*/*Os06g0275000*) between melon and rice. Additionally, three pairs of paralogous genes (*CmBBX4*/*CmBBX5*, *CmBBX3*/*CmBBX17*, *CmBBX19*/*CmBBX10*) were found within the melon BBX gene family. This suggests a certain degree of structural similarity among these genes, which may translate to analogous biological functions. Drawing from existing literature on the functions of similar genes in *Arabidopsis* and rice, potential biological roles of the melon BBX genes can be inferred.

### 2.4. Gene Structure and Conserved Motif Analysis of CmBBXs

**Figure 3:**
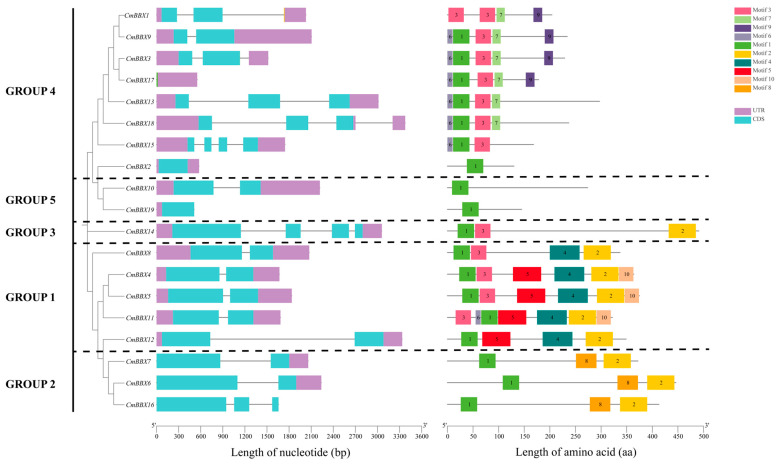
Gene structures and protein motifs of the melon BBX gene family.

Clustering analysis of the BBX gene family in melon, utilizing TBtools software (version 11.0.13) for visualization, revealed that the members can be classified into five subfamilies: GROUP1, GROUP2, GROUP3, GROUP4, and GROUP5 (Figure 3). This classification aligns closely with the clustering results observed in melon, *Arabidopsis*, and rice.

Structural analysis of the melon BBX family indicated that in GROUP3, the average number of exons and introns was 2.25 and 1.625, respectively, while GROUP5 exhibited averages of 1.5 exons and 0.5 introns. In contrast, the averages for GROUP1 were 2 exons and 1 intron, and for GROUP2, 2.33 exons and 1.33 introns. Genes within the same subfamily typically exhibit similarities in exon-intron structure. This structural homogeneity, alongside significant sequence homology, suggests that gene duplication events may have occurred during the evolution of the melon BBX gene family.

Conserved motif analysis identified ten conserved motifs within the melon BBX genes. Examination of the schematic diagram indicated that BBX genes within the same subfamily shared identical conserved sequences and similar arrangement patterns. This uniformity suggests that while motifs and arrangements may have diverged functionally among different subfamilies, the conserved motifs within the same subfamily likely indicate analogous biological functions.

### 2.5. Gene Duplication and Synteny Analysis

**Figure 4:**
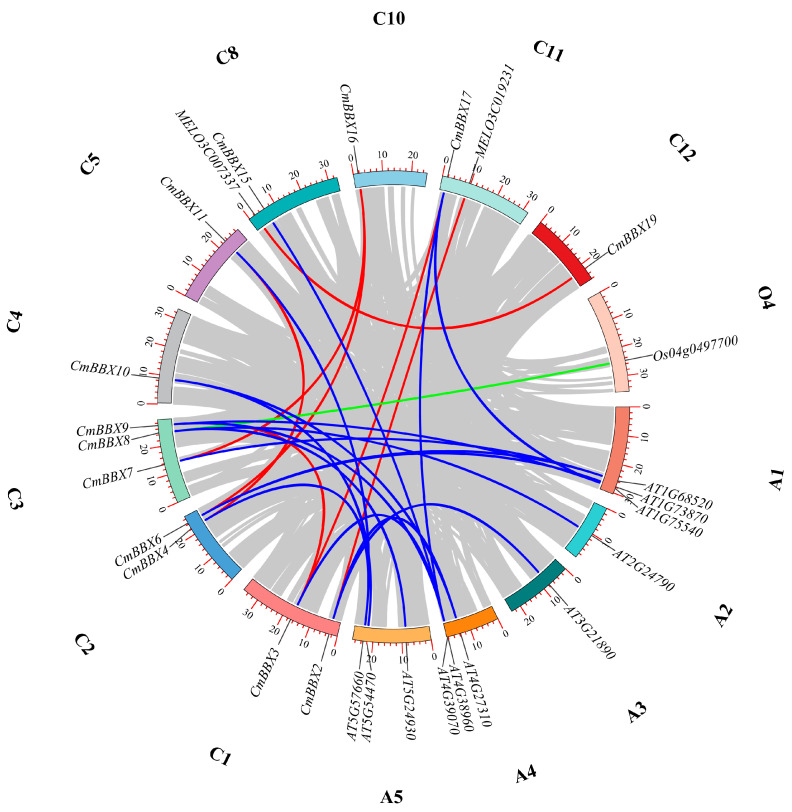
Assessment of the BBX gene duplication between melon, *Arabidopsis*, and rice. C denotes melon, A represents *Arabidopsis*, and O signifies rice, with the adjacent numbers indicating the corresponding chromosome numbers.

To further investigate the evolutionary aspects of the melon BBX gene family, we performed a collinearity analysis involving 19 melon BBX genes and their counterparts in *Arabidopsis* and rice (Figure 4). The analysis revealed collinearity among seven pairs of melon BBX genes: *CmBBX2*/*MELO3C019231*, *CmBBX3*/*CmBBX17*, *CmBBX3*/CmBBX9, *CmBBX4*/*CmBBX11*, *CmBBX6*/*CmBBX16*, *CmBBX7*/*CmBBX16*, and *MELO3C007337*/*CmBBX19*. Additionally, one melon BBX gene, *CmBBX8*, exhibited collinearity with a single rice gene, *Os04g0497700*.

Furthermore, 11 melon BBX genes demonstrated 17 collinearity relationships with 11 BBX genes from *Arabidopsis*, specifically: *AT1G75540*/*CmBBX17*, *AT1G68520*/*CmBBX7*, *AT1G75540*/*CmBBX9*, *AT1G73870*/*CmBBX6*, *AT1G68520*/*CmBBX6*, *AT2G24790*/*CmBBX8*, *AT3G21890*/*CmBBX2*, *AT4G27310*/*CmBBX2*, *AT4G39070*/*CmBBX17*, *AT4G27310*/*CmBBX10*, *AT4G39070*/*CmBBX3*, *AT4G39070*/*CmBBX9*, *AT4G38960*/*CmBBX15*, *AT5G57660*/*CmBBX4*, *AT5G54470*/*CmBBX10*, *AT5G57660*/*CmBBX11*, and *AT5G24930*/*CmBBX8*.

The remaining six melon BBX genes—*CmBBX12*, *CmBBX13*, *CmBBX1*, *CmBBX5*, *CmBBX14*, and *CmBBX18*—did not exhibit collinearity with those in melon, *Arabidopsis*, or rice. This observation suggests that these particular genes are relatively conserved within the BBX gene family.

### 2.6. Analysis of the Cis-Acting Elements in CmBBXs

**Figure 5:**
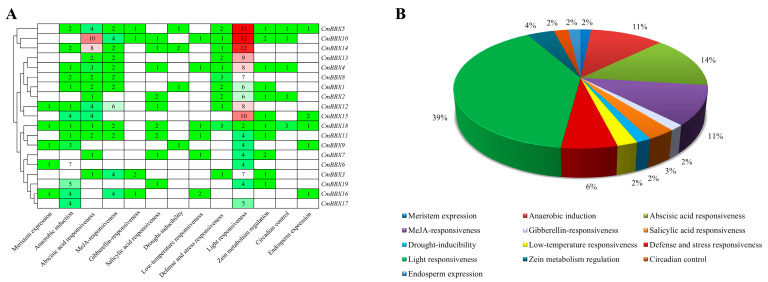
*Cis*-regulatory elements in the promoter region of *CmBBXs* (**A**) and their proportions (**B**). The numbers in (**A**) indicate the quantity of the element in the corresponding gene.

In our analysis of *cis*-regulatory elements, we identified 13 distinct elements within the promoter sequences of the BBX family genes in melon. The number of *cis*-regulatory elements varies among different genes; for instance, *CmBBX18* contains the highest diversity, encompassing a total of 11 elements, while *CmBBX17* has the fewest, with only 2 types (Figure 5A).

Among all identified *cis*-regulatory elements, the light responsiveness element is the most prevalent, constituting 39% of the total. The abscisic acid responsiveness element accounts for 14%, while both anaerobic induction and MeJA responsiveness elements each represent 11%. Additional elements include those related to stress responses, circadian control, endosperm expression, and meristem expression, among others (Figure 5B).

### 2.7. Tissue-Specific Expression Analysis of CmBBXs

**Figure 6:**
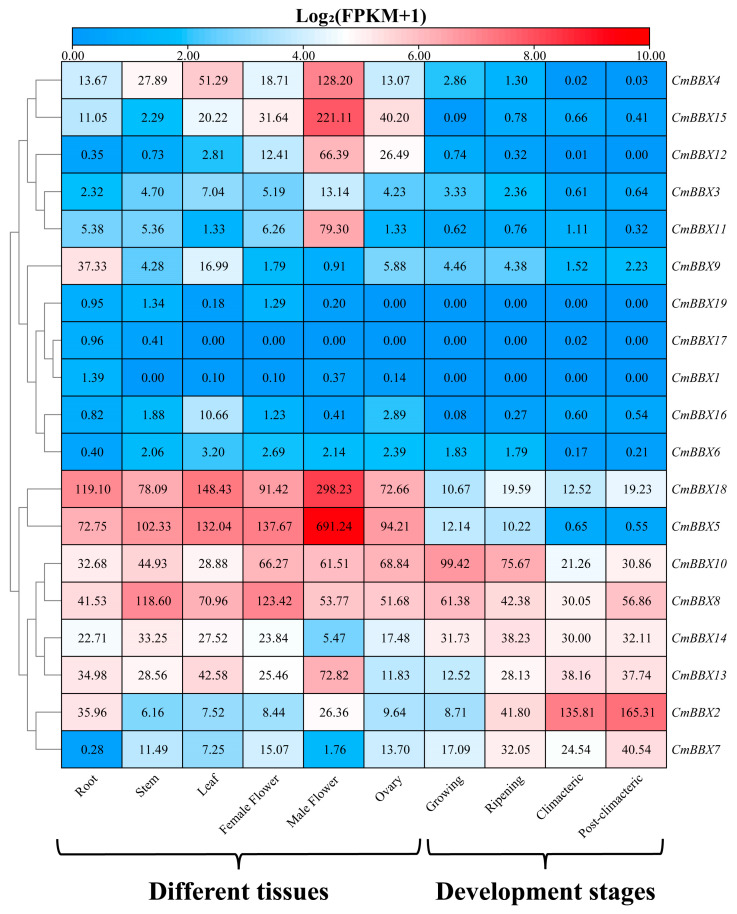
Expression analysis of *CmBBXs* in different tissues and development stages.

By re-analyzing published transcriptome data across various tissues and developmental stages of melon, alongside genomic data, we delineated the specific expression profiles of the BBX gene family (Figure 6). Tissue-specific analysis revealed that *CmBBX18*, *CmBBX5*, *CmBBX10*, and *CmBBX8* exhibited high expression levels in six organs, including roots, stems, and leaves. Notably, *CmBBX14* was highly expressed in all organs except male flowers, where its expression was low. In contrast, *CmBBX2* demonstrated elevated expression in roots and male flowers, while exhibiting reduced expression in other tissues. *CmBBX4* was specifically expressed in leaves and male flowers. Moreover, *CmBBX15* showed differential expression patterns in male flowers, female flowers, and the ovary, suggesting its potential involvement in the regulation of melon reproductive organs. Additionally, *CmBBX12* and *CmBBX11* were exclusively expressed in male flowers, and *CmBBX9* displayed significantly higher expression in roots compared to other organs.

Analysis of the expression profiles of melon BBX genes during various developmental stages revealed that *CmBBX10*, *CmBBX8*, and *CmBBX14* were highly expressed across all four stages of fruit development. *CmBBX13* and *CmBBX2* exhibited relatively low expression during the growing stage, whereas the remaining genes were highly expressed. *CmBBX7* showed specific expression during the ripening and post-climacteric stages, while other BBX genes demonstrated low expression levels or were not expressed.

### 2.8. Expression Patterns Analysis of CmBBXs Under Abiotic Stresses

**Figure 7:**
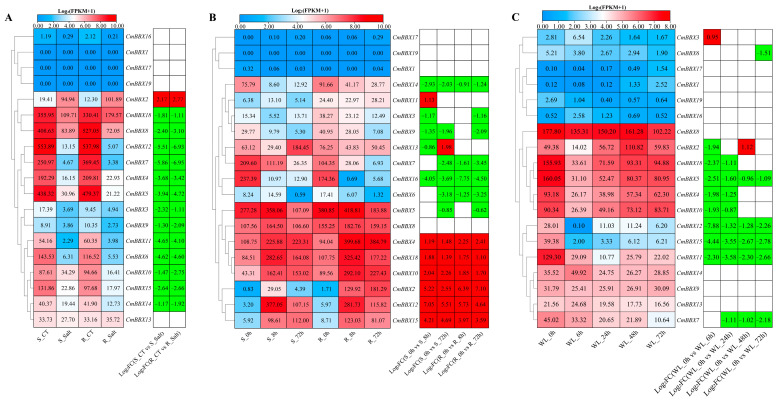
Abiotic stress expression profile of *CmBBXs*. S: susceptible plant; R: resistant plant. (**A**) The expression of salt stress. (**B**) The expression of cold stress. (**C**) The expression of waterlogging stress. The data in the left expression heatmaps are the original FPKM values; the data in the right boxes are log_2_ (fold change) values highlighted by red (upregulation) and green (downregulation) colors.

Utilizing transcriptome sequencing data from various melon varieties exposed to salt, cold, and waterlogging stress, in conjunction with genomic information, we analyzed the expression of BBX family genes under these abiotic stress conditions and generated a heatmap (Figure 7).

Under salt stress (Figure 7A), only the *CmBBX2* gene exhibited significant up-regulation in both susceptible and resistant materials compared to the control. Conversely, 13 genes—*CmBBX18*, *CmBBX8*, *CmBBX12*, *CmBBX7*, *CmBBX4*, *CmBBX5*, *CmBBX3*, *CmBBX9*, *CmBBX11*, *CmBBX6*, *CmBBX10*, *CmBBX15*, and *CmBBX14*—were significantly down-regulated in both categories of material.

In the context of cold stress (Figure 7B), *CmBBX14*, *CmBBX3*, *CmBBX9*, *CmBBX7*, *CmBBX16*, *CmBBX6*, and *CmBBX5* showed significant down-regulation in both susceptible and resistant materials relative to the control. Conversely, *CmBBX4*, *CmBBX18*, *CmBBX10*, *CmBBX2*, *CmBBX12*, and *CmBBX15* were significantly up-regulated across both material types. Notably, *CmBBX11* was only up-regulated in the susceptible material, while *CmBBX13* exhibited both up-regulation and down-regulation within this same group.

Under waterlogging stress (Figure 7C), compared to the control treatment, *CmBBX5*, *CmBBX12*, *CmBBX15*, and *CmBBX11* demonstrated significant down-regulation at 6, 24, 48, and 72 h of waterlogging treatment. *CmBBX7* showed significant down-regulation after 24, 48, and 72 h. Furthermore, *CmBBX18* and *CmBBX10* were significantly down-regulated at 6 and 24 h, while *CmBBX3* was up-regulated at 6 h. *CmBBX6* exhibited significant down-regulation after 72 h, and *CmBBX2* showed down-regulation at 6 h but up-regulation after 48 h of treatment.

### 2.9. Expression Patterns Analysis of CmBBXs Under Biotic Stresses

**Figure 8:**
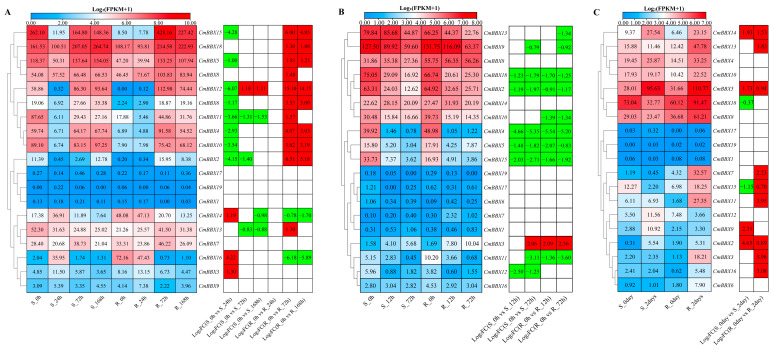
Biotic stress expression profile of *CmBBXs*. S: susceptible plant; R: resistant plant. (**A**) The expression of powdery mildew stress. (**B**) The expression of *Fusarium* wilt stress. (**C**) The expression of gummy stem blight stress. The data in the left expression heatmaps are the original FPKM values; the data in the right boxes are log_2_ (fold change) values highlighted by red (upregulation) and green (downregulation) colors.

To further investigate the expression patterns of BBX genes in melon under various biotic stresses, this study reanalyzed transcriptome data pertaining to powdery mildew, bacterial wilt, and stem canker, integrating this information with melon genomic data (Figure 8).

Under powdery mildew stress (Figure 8A), *CmBBX15*, *CmBBX5*, *CmBBX6*, *CmBBX11*, *CmBBX4*, *CmBBX10*, *CmBBX2*, and *CmBBX13* exhibited down-regulation in the resistant material, while they were significantly up-regulated in the susceptible material. In contrast, *CmBBX16* was up-regulated in the susceptible material and down-regulated in the resistant material. *CmBBX12* was up-regulated in the resistant material, showing both up-regulation and down-regulation in the susceptible material. Additionally, *CmBBX8* was exclusively up-regulated in the resistant material, while *CmBBX3* was only up-regulated in the susceptible material.

Under *Fusarium* wilt stress (Figure 8B), only *CmBBX3* demonstrated significant up-regulation in both resistant and susceptible materials compared to the control. The remaining seven genes (*CmBBX9*, *CmBBX18*, *CmBBX4*, *CmBBX5*, *CmBBX15*, and *CmBBX11*) exhibited significant down-regulation in both material types. Furthermore, *CmBBX13* and *CmBBX10* were down-regulated solely in the resistant material, and *CmBBX12* was down-regulated only in the susceptible material.

Under gummy stem blight stress (Figure 8C), 11 genes displayed significant differential expression relative to the control. Notably, *CmBBX14*, *CmBBX5*, and *CmBBX2* were significantly up-regulated in both resistant and susceptible materials. In contrast, *CmBBX18* and *CmBBX9* exhibited down-regulation and up-regulation, respectively, in the susceptible material. *CmBBX13*, *CmBBX7*, *CmBBX11*, *CmBBX3*, and *CmBBX16* were up-regulated exclusively in the resistant material. Remarkably, *CmBBX15* was down-regulated in the susceptible material while being up-regulated in the resistant material.

### 2.10. Regulation Patterns of CmBBXs Under Stresses

**Figure 9:**
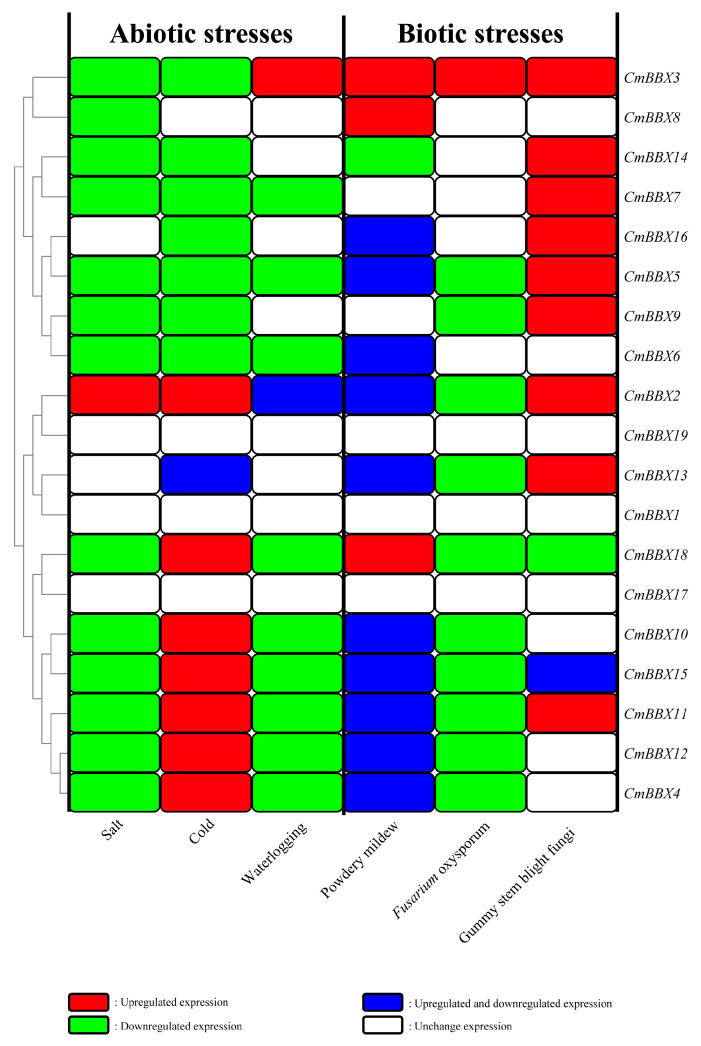
An expression pattern heatmap of the *CmBBXs* under abiotic and biotic stresses.

By examining the expression patterns of *CmBBXs* under various stress conditions, DEGs were identified, and corresponding expression heatmaps were generated (Figure 9). The analysis revealed that six genes—*CmBBX3*, *CmBBX5*, *CmBBX2*, *CmBBX18*, *CmBBX15*, and *CmBBX11* exhibited differential expression under both abiotic and biotic stresses, indicating their active involvement in the stress response of melons. Future studies should focus on these six genes. Conversely, other genes, such as *CmBBX7*, *CmBBX5*, and *CmBBX6*, displayed down-regulation under abiotic stress, while responses to biotic stress varied, with some genes not being expressed, some up-regulated, and others showing both up-regulation and down-regulation. Notably, *CmBBX19*, *CmBBX1*, and *CmBBX17* did not exhibit differential expression under either abiotic or biotic stress, suggesting these genes were not regulated by the above stresses.

### 2.11. RT-qPCR Analysis of the CmBBXs

**Figure 10:**
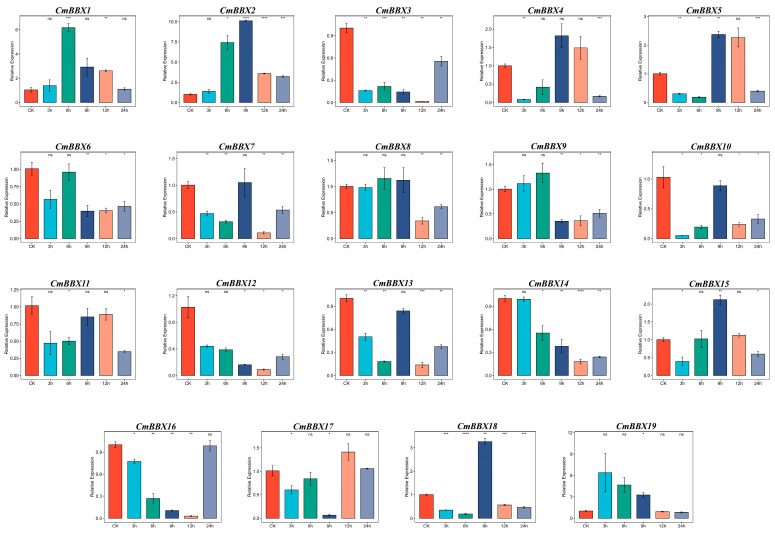
Expression levels of *CmBBXs* under salt treatments. Error bars are the average error of three biological replicates. Asterisks are used to indicate the significant degree of the expression level compared to the value of the control (* *p* < 0.05, ** *p* < 0.01, *** *p* < 0.001, **** *p* < 0.0001).

To investigate the expression pattern differences of BBX family genes in melons under salt stress and to validate the accuracy of the transcriptome data analysis in this study, we subjected the melon cultivar “TQ1” to a salt stress treatment (300 mM) at time points of 0 h, 3 h, 6 h, 9 h, 12 h, and 24 h. RT-qPCR was employed to assess the expression changes of 19 BBX genes (Figure 10). The transcriptome data corresponded to samples taken after 24 h of salt stress treatment. Comparison with RT-qPCR results showed that the expression patterns of the BBX genes were largely consistent. Analysis indicated significant changes in the expression levels of various BBX genes over the 0 to 24-h period, with notable differences observed. Specifically, *CmBBX1* and *CmBBX2* exhibited similar expression trends, initially increasing, followed by a subsequent decline. Additionally, *CmBBX12*, *CmBBX14*, and *CmBBX16* displayed comparable expression patterns, characterized by high expression at 0 h and a gradual decrease over time, with a significant increase after 24 h of treatment. Moreover, the expression levels of six genes—*CmBBX2*, *CmBBX4*, *CmBBX5*, *CmBBX7*, *CmBBX15*, and *CmBBX18*—were markedly higher after 9 h of treatment compared to other time points, suggesting their potential involvement in the regulatory response of melons to salt stress.

## 3. Discussion

The BBX gene functions as a prominent TF, playing a direct role in growth and development processes, encompassing signal transduction, floral organ formation, hormonal responses, and stress responses [29]. For example, Wu et al. [30] demonstrated that *BBX1* and *BBX13* in Saccharum significantly promote plant growth and enhance tolerance to nitrogen stress. Zhou et al. [31] identified that the *CaBBX14* gene in peppers responds to Phytophthora disease stress, with similar implications for cucurbit plants, such as cucumber [10] and watermelon [12]. There is a notable lack of related studies in melon. This gap significantly hampers the understanding of the biological functions of BBX genes in this species. This study employed the Hidden Markov Model file PF00643 to identify 19 BBX genes within the melon genome database. An analysis of the physicochemical properties, phylogenetic relationships, collinearity, gene structure, and expression patterns under stress conditions was conducted to investigate the evolution and potential functional differentiation of melon BBX genes. Chromosomal localization indicated that these 19 genes were unevenly distributed across 12 melon chromosomes, with no genes present on chromosomes 6 and 9. In the phylogenetic analysis, the 19 melon BBX genes were categorized into five subfamilies. Examination of gene structures and conserved motifs revealed a consistent arrangement within the same subfamily. Comparative analysis showed a pair of direct homologous genes between the melon BBX gene family and those of *Arabidopsis* and rice, suggesting a high degree of homology. Consequently, the biological functions of melon BBX genes can be inferred from their homologous counterparts in *Arabidopsis* and rice. Gene duplication analysis identified one tandemly duplicated gene and five fragmentally duplicated genes, indicating that large-scale gene amplification of BBX genes in melon is unlikely. This finding corroborates similar observations reported in studies of other plant gene families [32].

Currently, genomic research is intrinsically linked to transcriptomics, which is a vital component of genomics and increasingly favored by researchers [33]. Advances in high-throughput sequencing technology have significantly enhanced sequencing efficiency, while the emergence of additional sequencing companies has contributed to reduced costs [34]. To further investigate melons, an increasing number of agricultural researchers have undertaken transcriptome sequencing studies. Reports on melon-related transcriptomic research have risen progressively over the years, and these transcriptome datasets have undergone validation and peer review to gain broader recognition [35]. Consequently, effectively utilizing publicly available melon transcriptome data can lower research expenditures while enabling a more profound exploration of beneficial information, thereby offering enhanced insights into the molecular biological functions of melon genes [36].

Currently, numerous researchers are leveraging publicly available transcriptomic data to analyze the expression levels of the BBX gene family across various plant tissues. This approach not only elucidates the tissue-specific expression patterns of BBX genes, providing insights into their roles in growth, development, and organ formation, but also facilitates the identification of key genes involved in specific physiological processes through comparative analysis of expression levels in different tissues [37,38]. Such findings may serve as potential targets for breeding and genetic improvement initiatives. Ultimately, this knowledge will lay the groundwork for applications in plant functional genomics and related biotechnologies [39]. For instance, in watermelon, *ClBBX14* and *ClBBX15*/*16* exhibit specific expression in leaves [12]. In *Trichosanthes kirilowii*, a total of 17 genes display differential expression during flowering [40]. Similarly, several BBX genes in pineapple show high expression levels during flowering, suggesting their potential involvement in floral bud differentiation. Additionally, overexpression of *acBBX18* in *Arabidopsis* has been demonstrated to promote flowering [41]. In the present study, we analyzed the specificity and expression patterns of BBX family genes across various melon tissues and developmental stages of fruit. Notably, *CmBBX18*, *CmBBX5*, *CmBBX10*, and *CmBBX8* are highly expressed in all six tissues examined, indicating their direct involvement in the progression from seedling to ovary formation in melon [42]. Conversely, certain genes, such as *CmBBX12* and *CmBBX11*, display tissue-specific expression restricted to male flowers, while *CmBBX9* is specifically expressed in roots. Furthermore, *CmBBX10*, *CmBBX8*, and *CmBBX14* maintain high expression levels throughout the four stages of fruit development, suggesting their roles in regulating cell division, expansion, and the synthesis of endogenous hormones, thereby promoting fruit development and quality. This consistent expression pattern may also indicate their significant roles in responding to external environmental signals and internal regulatory pathways, providing crucial insights into the molecular mechanisms underlying fruit development in melon [43].

The mechanisms by which plants respond to environmental stressors have long been a focal point of research. Enhancing the adaptability of plants to adverse conditions is a primary objective for breeders. The BBX gene family, recognized as a significant group of transcription factors in plants, has been the subject of extensive studies. For instance, in soybean, the expression levels of 22 *GmBBX* genes significantly increased under salt stress, with each gene exhibiting more than a twofold enhancement, indicating their critical role in salt tolerance [11]. In *Salvia miltiorrhiza*, a total of seven BBX genes were found to be significantly upregulated in response to salt stress, while six BBX genes were upregulated under drought stress, suggesting their contribution to the plant’s adaptation to saline conditions [44]. In tobacco, genes such as *NtBBX8*, *NtBBX14*-*15*, and *NtBBX28* showed significant upregulation ten days post-inoculation with Ralstonia solanacearum, positively influencing normal growth and development [45].

To further investigate the role of *CmBBX* genes in melon under stress conditions, this study reanalyzed publicly available transcriptomic data in conjunction with the melon genome, focusing on the responses to salt, cold, flooding, powdery mildew, gummy stem blight, and *Fusarium* wilt. The analysis revealed that the majority of BBX genes were significantly downregulated under abiotic stress conditions, particularly in response to salt and waterlogging stress. For instance, under salt stress, 13 *CmBBX* genes exhibited significant downregulation in both resistant and susceptible materials. Similarly, during cold stress, 7 *CmBBX* genes were downregulated in both resistant and susceptible materials. Waterlogging stress resulted in the downregulation of 4 genes across all four time points post-treatment. This implies that these genes are subjected to negative modulation during mRNA transcription under stress conditions [46]. The associated gene functions may be repressed or rendered superfluous for expression; this downregulation could be intricately linked to mechanisms that inhibit particular biological pathways, orchestrate cellular differentiation, or mount responses to pathogenic invasion [47]. In relation to biotic stresses (powdery mildew, gummy stem blight, and *Fusarium* wilt), most genes were downregulated in resistant materials during powdery mildew stress, whereas they were upregulated in susceptible materials, indicating that varietal differences significantly influence resistance mechanisms. Under gummy stem blight stress, the majority of genes showed downregulation, while under *Fusarium* wilt stress, they exhibited upregulation. This differential expression of BBX genes likely enhances the capacity of melon to respond to biotic stressors [48]. Furthermore, the similarity in expression patterns of these genes may indicate a conserved function in the physiological adaptation of plants, warranting further investigation to elucidate their specific roles and regulatory mechanisms, thus providing a theoretical basis for improving crop resistance to diseases [49].

## 4. Materials and Methods

### 4.1. Identification and Chromosomal Distribution of Melon BBX Gene Family Members

Relevant literature was reviewed, and the HMM model file (PF00643) for the BBX gene family was downloaded from the InterPro database (https://www.ebi.ac.uk/interpro/, accessed 1 November 2024) [50]. Additionally, the protein sequence file for melon DHL92 (V4) was obtained from the Cucurbit Genomics database (http://cucurbitgenomics.org/v2/, accessed 1 November 2024), allowing for the construction of a local protein database for analysis [51]. Using the hmmersearch program from the HMMER software package (version 3. 0) [52], a threshold E-value of less than 1 × 10^−5^ was set to identify potential melon BBX gene IDs from the protein database. Corresponding protein sequence information was extracted based on the initially identified gene IDs, which were subsequently uploaded to Pfam [53], SMART [54], and NCBI [55] for domain validation. This process facilitated the determination of the members of the melon BBX gene family. The physicochemical properties of the identified melon BBX family members were analyzed using the online tool ExPASy (https://web.expasy.org/protparam/, accessed 1 November 2024) [56]. Finally, the distribution of the melon gene family on the chromosome was illustrated using TBtools software (version 11.0.13) [57].

### 4.2. Analysis of the BBX Gene Family Characteristics and Phylogenetic Evolution in Melons

The CDS file of the melon BBX family genes was uploaded to the GSDS website (https://gsds.gao-lab.org/, accessed on 3 November 2024) [58] for gene structure analysis. Default parameters were utilized in the online software MEME (https://meme-suite.org/meme/, accessed on 3 November 2024) to analyze the conserved motifs of melon BBX family proteins [59]. BBX protein sequence files from melon, *Arabidopsis*, and rice were imported into MEGA 11 (version 2.138) [60] to construct a phylogenetic tree using the neighbor-joining method with default settings. To identify the *cis*-regulatory elements located 2000 bp upstream of the start codon of the BBX family genes, PlantCARE (http://bioinformatics.psb.ugent.be/webtools/plantcare/html/, accessed on 3 November 2024) [61] was employed. Subsequently, all *cis*-elements were classified according to their functions and visualized using TBtools software.

### 4.3. Synteny Analysis of BBX Family Genes from Melon, A. thaliana, and Rice

The MCScanX software (utilizing default parameters) [62] was employed to analyze tandem repeat and segmental repeat genes within the BBX gene family of melon. Additionally, collinearity analysis of the BBX gene family was performed among melon, *Arabidopsis*, and rice. Visualization of the results was conducted using Circos software (version 0.69.9) [63].

### 4.4. Tissue-Specific Expression of Melon BBX Family Genes

Transcriptome data from various melon tissues (PRJNA803327) and developmental stages (PRJNA543288) [64] were obtained from the NCBI database. Utilizing the melon genome data DHL92 (V4), the transcriptome was re-analyzed, and the corresponding heatmaps were generated using TBtools software.

### 4.5. Analysis of Expression Patterns of Melon BBX Gene Family Under Various Stresses

We retrieved transcriptome-related data for melon experiencing various stressors from the NCBI database, including salt stress (PRJNA296827) [19], cold stress (PRJNA418901), waterlogging stress (PRJNA726294) [65], powdery mildew stress (PRJNA358655) [66], *Fusarium* wilt stress (PRJNA842515) [67], and gummy stem blight (PRJNA681992) [68]. Subsequently, we re-analyzed the transcriptome and differentially expressed genes by integrating the melon genome data DHL92 (V4), and generated the corresponding heat maps using TBtools software.

### 4.6. RT-qPCR Analysis of Melon Under Salt Stress

In this study, the melon variety “TQ1” utilized in the experiment was supplied by the Vegetable Research Institute of the Anhui Academy of Agricultural Sciences. At the five-leaf-one-heart stage of seedling development, healthy and uniformly growing plants were selected for treatment. The roots of the seedlings were immersed in a 300 mM sodium chloride solution for durations of 0, 3, 6, 9, 12, and 24 h. For each treatment, three seedlings with consistent growth status were selected, from which leaf samples were collected at the same positional node. These samples were immediately flash-frozen in liquid nitrogen for subsequent RNA extraction and reverse transcription. Total RNA was extracted using the HiPure Plant RNA Mini Kit (Magen Biotech, Shanghai, China), and cDNA synthesis was performed with the SMART kit (Takara Bio, Shiga, Japan), following the manufacturer’s protocols. Real-time quantitative RT-PCR (RT-qPCR) was carried out with SYBR Green qPCR Premix (Low ROX) on an IQ5 real-time PCR detection system (Bio-Rad, Hercules, CA, USA). *Actin3* (GenBank accession number XM_008449644.2) served as the reference gene (Table S1). The reaction mixture comprised 7 µL of ddH2O, 10 µL of 2 × Mix, 1 µL of cDNA, and 1 µL each of positive and negative primers, totaling 20 µL. The thermal cycling conditions were set to 95 °C for 30 s, followed by 40 cycles of 95 °C for 5 s and 60 °C for 34 s, concluding with a final extension at 72 °C for 10 s. Data from three biological replicates were analyzed using the 2^−∆∆Ct^ method and processed in Excel 2021. Statistical significance was evaluated with a *t*-test in SPSS 19.0, and GraphPad 9.0 Prism was used for data visualization.

## 5. Conclusions

Our study was the first identification of 19 BBX family genes within the melon genome. We performed an analysis of the physicochemical properties of these genes and mapped their chromosomal locations, revealing their distribution across chromosomes 1, 2, 3, 4, 5, 7, 8, 10, 11, and 12. Cluster analysis categorized these genes into five subfamilies, with shared structural similarities among members of the same subfamily. Collinearity analysis uncovered seven collinear relationships among the 19 melon BBX genes, alongside 17 collinear relationships with *Arabidopsis* and one with rice. The analysis of *cis*-acting elements indicated that the most prevalent type was the light-responsive element, followed by the abscisic acid response element. We also examined the expression patterns of melon BBX genes across various organs and stages of fruit development, revealing their cooperative role in regulating melon growth and fruit maturation. Additionally, the expression profiles under different stress conditions demonstrated that most genes displayed variable expression levels, particularly *CmBBX3*, *CmBBX5*, *CmBBX2*, *CmBBX18*, *CmBBX15*, and *CmBBX11*, which exhibited differential expression across all stressors. These observations suggest that these genes are integral to the growth processes of melons. RT-qPCR analysis indicated that melon BBX genes expressed at varying levels over time under salt stress, with some differences evident among treatments. These findings establish a foundation for further investigation into the melon BBX gene family and provide promising candidate genes for enhancing resistance breeding in melons.

## Figures and Tables

**Table 1:** Information and physicochemical properties of 19 *CmBBX* members.

Gene ID	CDS Size (bp)	Number of Amino Acid (aa)	Molecular Weight (kDa)	Theoretical pI	Instability Index	Aliphatic Index	Grand Average of Hydropathicity	Prediction of Subcellular Location
*MELO3C018684*/*CmBBX1*	615	204	22.70	5.96	43.51	66.57	−0.419	Nuclear
*MELO3C018801*/*CmBBX2*	393	130	14.57	7.48	34.68	76.46	−0.285	Extracellular
*MELO3C013430*/*CmBBX3*	690	229	24.92	6.12	50.08	65.33	−0.399	Nuclear
*MELO3C017501*/*CmBBX4*	1095	364	40.13	6.01	43.97	71.48	−0.302	Nuclear
*MELO3C017500*/*CmBBX5*	1128	375	41.10	5.96	44.18	67.04	−0.351	Nuclear
*MELO3C017304*/*CmBBX6*	1341	446	51.38	5.86	51.61	69.08	−0.708	Nuclear
*MELO3C011576*/*CmBBX7*	1119	372	42.68	8.35	55.04	66.80	−0.8	Nuclear
*MELO3C011317*/*CmBBX8*	1014	337	36.88	6.08	43.97	69.23	−0.494	Nuclear
*MELO3C010985*/*CmBBX9*	705	234	24.95	6.69	48.46	85.85	−0.051	Extracellular
*MELO3C018174*/*CmBBX10*	825	274	30.04	4.35	61.29	52.66	−0.839	Nuclear
*MELO3C004050*/*CmBBX11*	969	322	35.47	8.22	51.92	65.47	−0.397	Nuclear
*MELO3C018930*/*CmBBX12*	1050	349	39.05	5.23	43.96	61.26	−0.805	Nuclear
*MELO3C016115*/*CmBBX13*	894	297	32.36	5.22	55.88	62.42	−0.452	Nuclear
*MELO3C017755*/*CmBBX14*	1476	491	54.32	5.58	42.34	67.54	−0.56	Nuclear
*MELO3C007886*/*CmBBX15*	507	168	18.85	6.59	55.32	72.50	−0.576	Extracellular
*MELO3C012248*/*CmBBX16*	1242	413	46.28	5.51	41.04	63.34	−0.708	Nuclear
*MELO3C023341*/*CmBBX17*	537	178	19.44	7.01	62.73	75.11	−0.25	Nuclear
*MELO3C022445*/*CmBBX18*	714	237	26.05	4.95	43.37	74.14	−0.29	Extracellular
*MELO3C002548*/*CmBBX19*	438	145	16.14	5.58	55.01	65.86	−0.559	Nuclear

## Data Availability

The original contributions presented in this study are included in the article/Supplementary Material. Further inquiries can be directed to the corresponding author Xiaomin Lu (luxm@ahstu.edu.cn).

## Supplementary Materials

The following supporting information can be downloaded at: https://www.mdpi.com/article/10.3390/plants14172715/s1, Table S1: Primers used for RT-qPCR.
